# Effect of nitric oxide on mitochondrial activity of human synovial cells

**DOI:** 10.1186/1471-2474-12-42

**Published:** 2011-02-08

**Authors:** Berta Cillero-Pastor, Miguel A Martin, Joaquín Arenas, María J López-Armada, Francisco J Blanco

**Affiliations:** 1 Osteoarticular and Aging Research Unit, Biomedical Research Center, INIBIC, CH Universitario da Coruña. Xubias 84, 15006, A Coruña, Spain; 2 Laboratorio de Investigación, Hospital 12 de Octubre, Madrid, Spain; 3 Inflammation Unit, Biomedical Research Center, INIBIC, CH Universitario da Coruña, Xubias 84, 15006, A Coruña, Spain

## Abstract

**Background:**

Nitric oxide (NO) is a messenger implicated in the destruction and inflammation of joint tissues. Cartilage and synovial membrane from patients with rheumatoid arthritis (RA) and osteoarthritis (OA) have high levels of NO. NO is known to modulate various cellular pathways and, thus, inhibit the activity of the mitochondrial respiratory chain (MRC) of chondrocytes and induce the generation of reactive oxygen species (ROS) and cell death in multiple cell types. For these reasons, and because of the importance of the synovial membrane in development of OA pathology, we investigated the effects of NO on survival, mitochondrial function, and activity of fibroblastic human OA synovial cells.

**Methods:**

Human OA synovia were obtained from eight patients undergoing hip joint replacement. Sodium nitroprusside (SNP) was used as a NO donor compound and cell viability was evaluated by MTT assays. Mitochondrial function was evaluated by analyzing the mitochondrial membrane potential (Δψm) with flow cytometry using the fluorofore DePsipher. ATP levels were measured by luminescence assays, and the activities of the respiratory chain complexes (complex I: NADH CoQ_1 _reductase, complex II: succinate dehydrogenase, complex III: ubiquinol-cytochrome c reductase, complex IV: cytochrome c oxidase) and citrate synthase (CS) were measured by enzymatic assay. Protein expression analyses were performed by western blot.

**Results:**

SNP at a concentration of 0.5 mM induced cell death, shown by the MTT method at different time points. The percentages of viable cells at 24, 48 and 72 hours were 86.11 ± 4.9%, 74.31 ± 3.35%, and 43.88 ± 1.43%, respectively, compared to the basal level of 100% (*p < 0.05). SNP at 0.5 mM induced depolarization of the mitochondrial membrane at 12 hours with a decrease in the ratio of polarized cells (basal = 2.48 ± 0.28; SNP 0.5 mM = 1.57 ± 0.11; *p < 0.01). The time course analyses of treatment with SNP at 0.5 mM demonstrated that treatment reliably and significantly reduced intracellular ATP production (68.34 ± 14.3% vs. basal = 100% at 6 hours; *p < 0.05). The analysis of the MRC at 48 hours showed that SNP at 0.5 mM increased the activity of complexes I (basal = 36.47 ± 3.92 mol/min/mg protein, SNP 0.5 mM = 58.08 ± 6.46 mol/min/mg protein; *p < 0.05) and III (basal = 63.87 ± 6.93 mol/min/mg protein, SNP 0.5 mM = 109.15 ± 30.37 mol/min/mg protein; *p < 0.05) but reduced CS activity (basal = 105.06 ± 10.72 mol/min/mg protein, SNP at 0.5 mM = 66.88 ± 6.08 mol/min/mg protein.; *p < 0.05), indicating a decrease in mitochondrial mass. Finally, SNP regulated the expression of proteins related to the cellular cycle; the NO donor decreased bcl-2, mcl-1 and procaspase-3 protein expression.

**Conclusions:**

This study suggests that NO reduces the survival of OA synoviocytes by regulating mitochondrial functionality, as well as the proteins controlling the cell cycle.

## Background

Osteoarthritis (OA) is a common cartilage and joint disease related to age and characterized by a reduction in the number of chondrocytes, loss of the extracellular matrix, and synovial inflammation [1,2]. It has been shown that in the last phases of OA the synovial membrane plays an important role in the progression of the pathology. This tissue synthesizes inflammation mediators, such as cytokines [interleukin-1α (IL-1α), IL-1β and tumor necrosis factor-α (TNF-α)], proteases (collagenases and the aggrecanases), lipidic mediators [prostaglandin E2 (PGE2) and leukotriene B4 (LTB4)], and nitric oxide (NO) [3].

NO is a small hydrophobic molecule with chemical properties that make it uniquely suitable as both an intra- and intercellular messenger [4]. NO is produced in high quantities by the synovium and chondrocytes in rheumatoid pathologies, such as OA and rheumatoid arthritis (RA) [5-8]. Recent studies show that NO influences mitochondria, particularly in the activity of the mitochondrial respiratory chain (MRC). NO has many consequences on cell function, including cell death [9,10]. The mitochondrion is a complex organelle that, depending on the tissue type, has variable functions in cellular processes, such as controlling the oxidative state of the cell [11,12]. In addition, the mitochondrion plays an important role in energy production, predominantly in vascularised aerobic tissues, as a generator of ATP. The mitochondrion also regulates caspase-dependent and caspase-independent apoptotic pathways [13]. The classical signals for programmed cell death are preceded by mitochondrial alterations, which include loss of mitochondrial membrane potential (ΔΨ), decrease in energy production, increase in the permeability of the mitochondrial membrane, alteration of MRC activities, release of pro-apoptotic factors, such as cytocrome c and downregulation of antiapoptotic members, such as bcl-2 and mcl-1, or activation of caspases pathways [14,15].

A variety of NO donors suppress the mitochondrial respiration in different cell types, affecting energy production [11,12]. Our study demonstrates that sodium nitroprusside (SNP), a NO donor compound, reduces activity of complex IV of the MRC of chondrocytes, causing apoptotic cell death [10,16]. Because synovial cells are also affected in OA pathology, expressing high levels of iNOs and producing high quantities of NO [17-19], and because mitochondria may play a role in this highly vascular aerobic tissue, we examined the effects of SNP on synoviocyte survival relative to mitochondrial function.

## Methods

### Procurement, processing and culture of synoviocytes

Human OA synovia were obtained from patients undergoing hip joint replacement surgery at the Orthopaedic Department of the Complejo Hospitalario Universitario da Coruña. Human OA synovial explants were enzymatically digested with 1.25 mg/ml bovine pancreas trypsin (Roche Diagnostics, Mannheim, Germany) for one hour with mixing at 37°C. The cells were then centrifuged and resuspended in RPMI medium (BioWhittaker, Verviers, Belgium) enriched with 20% fetal calf serum (FCS) (Invitrogen, Carlsbad, CA, USA) and supplemented with 100 μl/ml glutamine (BioWhittaker), 100 μl/ml insulin-transferrin-sodium selenite (Sigma-Aldrich, St Louis, MO, USA). Culture was at 37°C in 5% CO_2 _in a 162 cm^2 ^flask (Costar, Cambridge, MA, USA) for four passages. After confluency was achieved, the cells were made quiescent by incubation for 24 hours in RPMI medium with 0.5% FCS before transfer to RPMI without FCS for the experiments. For the experiments, the cells were treated with the NO donor, SNP (Alexis Bio-chemicals, San Diego, CA, USA) at 0.5, 1 or 2 mM for varying times of incubation, or with valinomycin at 1 μM for the depolarization experiments (Sigma-Aldrich). Controls were cultured under basal conditions, RPMI only. This study was approved by the Ethics Committee of Galicia-Spain.

### Cell viability MTT assay

Cell viability (12 × 10^3 ^cells per well in a 96-well plate) was evaluated using a colorimetric MTT assay measuring reduction power (Roche Diagnostics). Following incubation of cells for different time points under basal conditions or with the experimental SNP concentrations, soluble tetrazolium salt solution (10 μl at 5 mg/ml in PBS) was added to the wells containing 100 μl of medium and the plate was incubated for an additional 4 hours. 100 μl of 10% SDS in 0.01 M HCl solubilization solution was then added to dissolve the water-insoluble formazan salt. Quantification with a spectrophotometer reader at 570 nm (GE Healthcare, Buckinghamshire, UK) was conducted.

### Determination of mitochondrial membrane potential (Δψm)

To measure the Δψm of synoviocytes, DePsipher (R&D, Abingdon, UK), a lipophilic cation (5,5',6,6'-tetrachloro-1,1',3,3'-tetraethylbenzimidazolyl carbocyanide iodide) was used. DePsipher exists as a monomer at low values of Δψm (green fluorescence) and forms aggregates at high Δψm (red fluorescence). Mitochondria with a normal Δψm concentrate DePsipher into aggregates (red fluorescence), but with a de-energized or depolarized Δψm, DePsipher forms monomers (green fluorescence). For this procedure, synoviocytes were seeded at 25 × 10^4 ^cells per well in 6-well plates in serum-free medium (basal conditions) or treated with the different stimuli for 12 hours. Valinomycin at 1 μM was utilized for the positive control. The synoviocytes collected by trypsinization were then incubated with 1 μl/ml DePsipher for 20 minutes at 37°C in 5% CO_2_, sedimented, washed in PBS and analyzed by flow cytometry using FACScan (Becton Dickinson, Montain View, CA, USA) and Cell-Quest software (Becton Dickinson). The analyzer threshold was adjusted on the forward light scatter (FSC) channel to exclude most subcellular debris. Photomultiplier settings were adjusted to detect green fluorescence DePsipher monomer signals, centered at ~390 nm, on the FL1 detector, and red fluorescence DePsipher aggregate fluorescence signals, centered at ~340 nm, on the FL2 detector. Mean fluorescence intensity values for FL1 and FL2, expressed as arbitrary units, were obtained for all experiments. In each experiment, at least 20,000 events were analyzed. The percentage of depolarization in a cell population was calculated by dividing the values of red fluorescence by the values of green fluorescence.

### Measurement of synoviocyte ATP levels

To determine intracellular ATP levels, a luminescent ATP detection assay system, based on the production of light from the reaction of ATP with luciferase and D-luciferin, was employed (Perkin Elmer, Barcelona, Spain). Synoviocytes were seeded at 5 × 10^4 ^cells per well in 96-well plates. Following incubation with or without SNP at 0.5 mM, 50 μl per well of mammalian cell lysis solution was added and the plate was shaken for five minutes in an orbital shaker. 50 μl of a substrate solution was then added and the microplate was shaken for another five minutes. The plate was dark-adapted for 10 minutes, and the emitted light was measured by luminometry in a Micro Beta TriLux (Perkin Elmer).

### Measurement of MRC complex activities in digitonin-permeabilized synoviocytes

Synoviocytes (3.5 × 10^6 ^per 140 mm diameter Petri dish) were stimulated with SNP at 0.5 mM for 48 hours. Synoviocytes grown in basal medium served as controls. The cells were collected by trypsinization, washed with phosphate buffered saline (PBS) and centrifuged at 150x*g *for five minutes at 4°C. The pellet was resuspended in 2 ml of ice-cold solution containing 20 mM MOPS, 0.25 M sucrose and 200 μg of digitonin per 5 × 10^6 ^cells. After five minutes incubation on ice, the suspension was centrifuged at 5,000 × *g *for three minutes at 4°C. The resulting pellet was resuspended in 1.5 ml of 20 mM MOPS, 0.25 M sucrose and 1 mM of EDTA buffer, incubated for five minutes and centrifuged at 10,000 × *g *for three minutes at 4°C. Finally, the pellet was resuspended in 200 μl 10 mM phosphate buffer (KP, pH 7.4), frozen and thawed once, then mildly sonicated. These digitonin-permeabilized homogenates were used to measure the activities of respiratory chain enzymes and CS using a DU-650 spectrophotometer (Beckman Instruments, Palo Alto, CA). Incubation temperatures were 30°C for rotenone-sensitive NADH-coenzyme Q_1 _reductase (complex I), succinate dehydrogenase (SDH; complex II), antimycin-sensitive ubiquinol cytochrome *c *reductase (complex III) and CS, and 38°C for cytochrome *c *oxidase (complex IV). To correct for mitochondrial volume, enzyme activities were normalized to the specific activity of CS. Complex I was measured by the oxidation of NADH at 340 nm in 20 mM KP (pH 8.0), 200 μM NADH, 1 mM NaN_3_, 0.1% bovine serum albumin (BSA)-EDTA, and 100 μM ubiquinone-1 (Sigma), first without rotenone (Calbiochem, La Jolla, CA, USA), then with 5 μM rotenone added so a rotenone-sensitive rate of NADH oxidation could be calculated. Complex II (SDH) activity was assessed by the reduction of 2,6-dichlorophenolindophenol (DCPIP) (Sigma) at 600 nm in 50 mM Tris-KP (pH 7.0), 1.5 mM KCN, 100 μM DCPIP, and 32 mM succinate (Sigma). Complex III was assayed by measuring the reduction of cytochrome *c *at 550 nm in 50 mM KP (pH 7.5), 2 mM NaN_3_, 0.1% BSA-EDTA, 50 μM cytochrome *c *(Roche) and 50 μM decyl-ubiquinol (Sigma), first without antimicyn A (Sigma) and then with 0.01 mg/ml of antimycin A added, so an antimicyn A-sensitive rate of cytochrome *c *reduction could be calculated. Complex IV was measured by the oxidation of reduced cytochrome *c *at 550 nm in 10 mM KP (pH 7.0) and 80 μM reduced cytochrome, which was freshly prepared before each experiment by adding sodium dithionite (Sigma). CS was evaluated at 420 nm in 75 mM Tris-HCl (pH 8), 100 μM 5,5'-dithiobis-(2-nitrobenzoic) acid (Sigma), 350 μg/ml acetyl-coenzyme A (Sigma), 0.5 mM oxaloacetate (Roche Diagnostics) and 0.1% Triton X-100 (Sigma).

### Western blot

Cells (1.5 × 10^6 ^per 100 mm diameter Petri dish) were incubated with SNP at 2 mM for 48 hours or in basal conditions. After incubation, the cells were washed in ice-cold PBS pH 7.5 and lysed in 0.2 M Tris-HCl pH 6.8 containing 2% SDS, 20% glycerol, 1 μg/ml cocktail inhibitor and 1 mM phenyl methyl sulfonyl fluoride (PMSF) (Sigma). Samples were boiled for 5 minutes and protein concentrations were determined using the BCA reagent assay (Pierce Chemical Co., Rockford, IL, USA). Thirty μg of the protein extract were resolved on 12.5% SDS-polyacrylamide gels, then transferred to polyvinylidene difluoride membranes (Immobilon P, Millipore Co., Bedford, MA, USA). These membranes were first blocked in Tris buffered saline (TBS) pH 7.4, containing 0.1% Tween-20 (Invitrogen) and 5% nonfat dried milk for 60 minutes at room temperature, then incubated overnight with mouse anti-human bcl-2 1:2000 (R&D), mouse anti-human mcl-1 1:500 (Oncogene, Cambridge MA, US) or mouse anti-human caspase 3 1:2000 (R&D) in fresh blocking solution at 4°C. After washing, the membranes were incubated with peroxidase conjugated secondary antibodies, developed using the ECL chemiluminescence kit (GE Healthcare) and digitized using LAS 3000 image analyzer. Quantitative changes in band intensities were evaluated using ImageQuant 5.2 software (GE Healthcare). Densitometric values for the Western blot bands containing the protein of interest were normalized against those of α-tubulin (Sigma) obtained from the same sample.

### Statistical analyses

The data are expressed as the mean ± SE from the determinations as described. Individual donors were studied in duplicate; pooled cells from different donors were not used in any experiment. The statistical software program, SPSS (version 15.0, SPSS, Chicago, IL, USA) was used to perform the analysis of variance (ANOVA) and Tukey tests. Differences were considered to be statistically significant at *p *≤ 0.05.

## Results

### Effect of NO on synoviocyte viability

**Figure 1:**
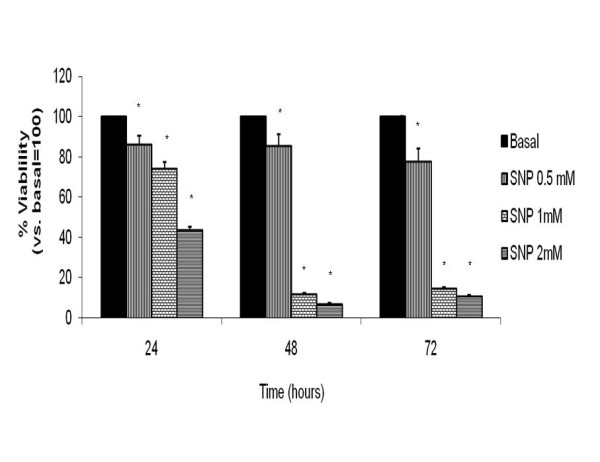
**Effect of sodium nitroprusside (SNP) on the cell viability of human osteoarthritic (OA) synoviocytes**. Human OA synoviocytes were incubated under basal conditions (RPMI only) or with SNP at concentrations of 0.5, 1 and 2 mM for 24, 48 and 72 hours. Cell viability was evaluated using a colorimetric analysis based on the MTT assay as detailed in the Methods section. The data are expressed as percentages of those of the control conditions (100%), and represent the mean ± standard error of eight different experiments performed in duplicate (*p < 0.05).

Analyses of viability using MTT assay methods to measure the cellular reduction power showed that the NO donor, SNP, reduced synoviocyte viability with a time-dependent decrease in the percentages (figure 1). At 24, 48 and 72 hours of incubation, respectively: SNP at 0.5 mM: 86.11 ± 4.9%, 74.31 ± 3.35%, 43.88 ± 1.43% vs. 100% basal; SNP at 1 mM: 85.52 ± 5.91%, 11.91 ± 0.72%, 7.1 ± 0.41% vs. 100% basal; and SNP at 2 mM: 77.85 ± 6.46%, 15 ± 0.52%, 10.85 ± 0.56% vs. basal 100%, ([n = 8]; *p < 0.05).

### Effect of SNP on mitochondria

**Figure 2:**
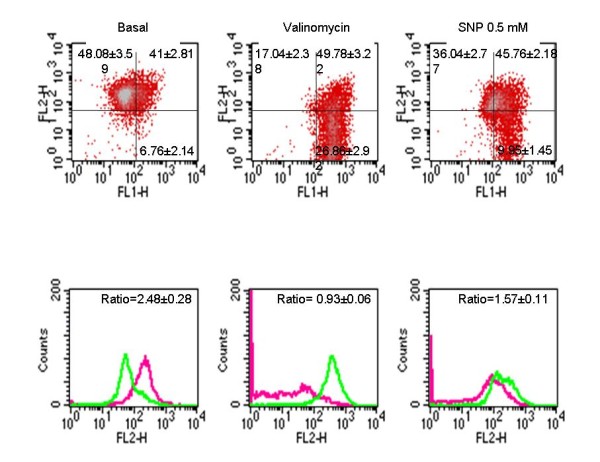
**Measurement of mitochondrial membrane depolarization following sodium nitroprusside (SNP) treatment of human osteoarthritic (OA) synoviocytes**. A) Cells were incubated for 12 hours in medium alone (control), with 0.5 mM SNP, or with 1 μM valinomycin (positive control). The mitochondrial membrane potential was determined by flow cytometry using DePsipher. Data acquisition was performed using a FACScan flow cytometer as detailed in the Methods section. A representative density plot for each treatment group is shown. The numbers represent the percentage of the mean ± standard error of each population from eight different experiments performed in duplicate (*p < 0.01). B) Histograms represent DePsipher fluorescence of SNP-stimulated synoviocytes. Relative to the control, green fluorescence (light line) increases while red fluorescence (dark line) decreases in SNP- or valinomycin-stimulated synoviocytes. The numbers represent the mean ± standard error of red/green fluorescence ratio values of eight different experiments performed in duplicate (*p < 0.01).

A second set of experiments examined the effects of SNP on mitochondrial function by determining the Δψm, ATP synthesis and activities of the respiratory chain complexes I-IV and CS. To assess the effect of SNP on the Δψm of synoviocytes, the fluoresecent probe DePsipher was used. The staining pattern of DePsipher for basal synoviocytes was established as the standard. The total cell population was divided into two subsets: one with normal Δψm (red fluorescence or high levels of FL-2), as the left upper panels represent in figure 2A, and another with lower Δψm (green fluorescence or high levels of FL-1), as the right lower panels represent in figure 2A. The treatment of synoviocytes with SNP at 0.5 mM for 12 hours reduced the percentage of cells with normal mitochondrial polarization [basal: 48.08 ± 3.59%; SNP at 0.5 mM: 36.04 ± 2.77%; and valinomycin at 1 μM: 17.04 ± 2.38% (n = 8, *p < 0.01)] (Figure 2A) and increased the number of cells with mitochondrial depolarization [basal: 6.76 ± 2.14%; SNP at 0.5 mM: 9.96 ± 1.45%; and valinomycin at 1 μM: 26.87 ± 2.92%, (n = 8, *p < 0.01)] (Figure 2A). SNP treatment at 0.5 mM caused a significant decrease in the red/green fluorescence ratio (basal: 2.48 ± 0.28; SNP at 0.5 mM: 1.57 ± 0.11; and valinomycin at 1 μM: 0.93 ± 0.06, (n = 8, *p < 0.01)] (figure 2B).

**Figure 3:**
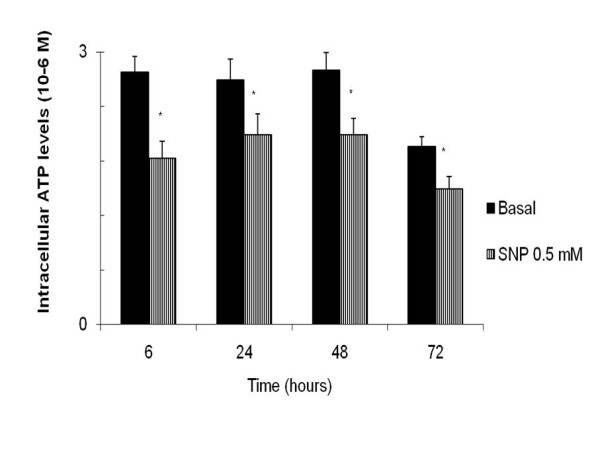
**ATP production of synoviocytes stimulated with sodium nitroprusside (SNP)**. Synoviocytes were cultured in basal conditions (only RPMI) or treated with SNP at 0.5 mM for 6, 24, 48 and 72 hours and collected for whole cell ATP measurement as described in the Methods section. ATP values are expressed as the mean ± standard error of eight different experiments performed in duplicate (*p < 0.05).

**Figure 4:**
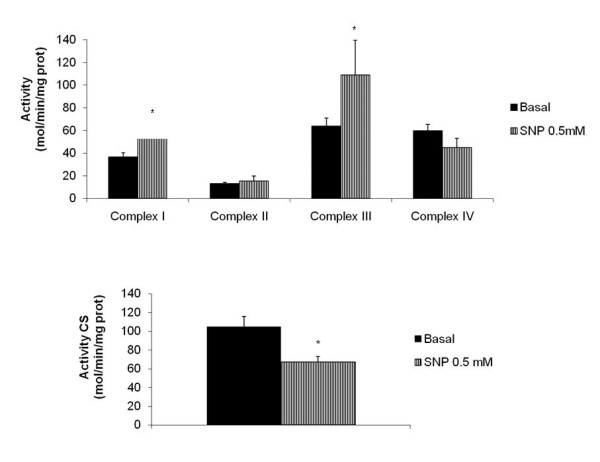
**Activity of mitochondrial respiratory chain (MRC) complexes of osteoarthritic (OA) synoviocytes incubated with sodium nitroprusside (SNP)**. A) Confluent synoviocytes were incubated for 48 hours under basal conditions (RPMI only) or with SNP at 0.5 mM and MRC complex activities were measured as reported in Methods. Citrate synthase (CS)-corrected complex activity is expressed as nmol/min/mg protein/CS specific activity x100. Complex I = rotenone-sensitive NADH-coenzyme Q1 reductase; Complex II = succinate dehydrogenase; Complex III = antimycin-sensitive ubiuqinol cytocrome c reductase; Complex IV = cytocrome c oxidase. The values are the mean ± standard error of six experiments performed in duplicate (*p < 0.05). B) The activity of CS following stimulation with SNP at 0.5 mM was evaluated. The values are the mean ± standard error of six experiments performed in duplicate (*p < 0.05).

We also assessed the effects of SNP on ATP production. Treatment with SNP at 0.5 mM for 6, 24, 48 and 72 hours significantly decreased ATP production [6 hours: 68.34 ± 14.3%; 24 hours: 75.04 ± 13.32%; 48 hours: 75.28 ± 12.94%; and 72 hours: 75.54 ± 16.3%, compared to the basal value of 100%, (n = 8) *p < 0.05)] (figure 3). These results indicate that SNP affects mitochondrial function, therefore, we evaluated the MRC activity of human OA synoviocytes treated with SNP at 0.5 mM for 48 hours. Figure 4 shows that the NO donor induced an increase in activity of complex I (basal: 36.47 ± 3.92 mol/min/mg prot.; SNP at 0.5 mM: 58.08 ± 6.46 mol/min/mg prot.) and of complex III (basal: 63.87 ± 6.93 mol/min/mg prot.; SNP at 0.5 mM: 109.15 ± 30.37 mol/min/mg prot.), (n = 6, *p < 0.05). However, SNP at 0.5 mM produced a decrease in the activity of CS [basal: 105.06 ± 10.72 mol/min/mg prot.; SNP at 0.5 mM: 66.88 ± 6.08 mol/min/mg prot., (n = 6, *p < 0.05)], which indicates a reduction in mitochondrial mass.

### Effect of SNP on cell cycle protein expression

**Figure 5:**
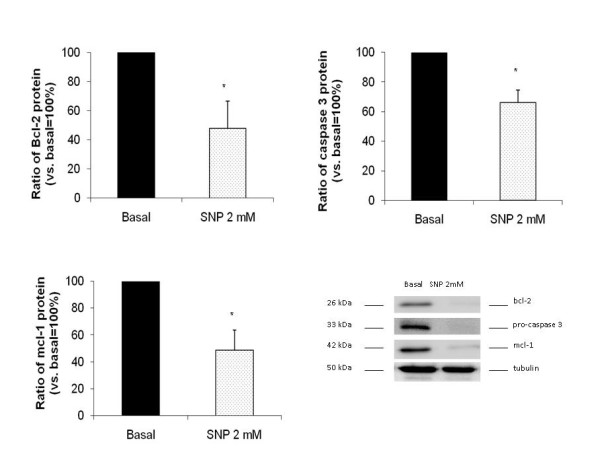
**Protein expression of apoptotic factors**. The expression of pro- and anti-apoptotic proteins was studied in synoviocytes treated with SNP at 2 mM for 48 hours. Cell protein lysates were subjected to western blot analysis. α-tubulin was used to standardize the total protein charged and expressions of bcl-2 (A) mcl-1 (B) and pro-caspase 3 (C) were evaluated. D) A representative experiment is shown. The results are shown as percentage of non-treated control cells (100%). Values are expressed as the mean ± standard error of three independent experiments (*p < 0.05).

Mitochondria are one of the initiators of apoptosis and regulate the cell cycle because they modulate the activity of anti- or pro-apoptotic elements, such as bcl-2 and the caspase family. To define the proteins implicated in SNP mitochondrial regulation and in induced death, we studied the expression pattern of bcl-2 and mcl-1 anti-apoptotic proteins, as well as the pro-apoptotic caspase 3. We observed that levels of bcl-2 protein in OA synoviocytes were downregulated by treatment with 2 mM SNP at 48 h [(47.75 ± 18.93% vs. basal 100%, (n = 4; *p < 0.05)] (figure 5A), as well those of mcl-1 [48.67 ± 15.00% vs. basal 100%, (n = 3; *p < 0.05] (figure 5B). We also found a decrease in the inactive proform of caspase 3 detected after treatment with SNP at 2 mM for 48 hours [66.02 ± 8.64 vs. basal 100%, (n = 3); *p < 0.05) (figure 5C)]. A representative experiment of each analysis is shown in figure 5D.

## Discussion

In OA pathology, the process leading to an aberrant cartilage structure characterized by reduction in the number of chondrocytes, loss of extracellular matrix, synovial inflammation, and irregular proliferation and death of synoviocytes is time- or age-dependent [20-22]. The pathogenesis of OA includes increased NO levels by chondrocytes and synovial cells as a consequence of the up-regulation of NO synthase (NOS) induced by IL-1β, TNF-α and other factors [23]. The role of NOS has been of interest in the pathogenesis of OA because of its role in the induction of chondrocyte and synoviocyte death [10]. Our earlier study established that the NO donor, SNP, induced an increase in hypodiploid DNA of human chondrocytes. It also induced mRNA expression of caspase-7 and reduced the expression of mRNA and protein synthesis of bcl-2 by human chondrocytes [10]. Further, SNP reduced chondrocyte survival and induced cell death with morphologic changes characteristic of chondrocyte apoptosis.

Mitochondria are important organelles in most cells and, particularly, in aerobic tissues, such as the synovial membrane. Pertaining to the importance of mitochondria in OA pathology, Rego et al. found that some haplogroups of mitochondrial respiratory genes of chondrocytes confer increased predisposition to the development of OA [24]. Also, the activity of the mitochondrial complexes II and III is lower in OA than in normal human chondrocytes. This produces a decrease in ATP levels as well as a higher reactive oxygen species (ROS) generation [25,26]. Mitochondrial complex inhibitors, such as antimycin A and oligomycin, induce ROS production, NF-κB activation, COX expression and PGE2 production in chondrocytes in culture [27]. In rabbit and human chondrocytes, SNP suppresses mitochondrial respiration by reducing oxygen consumption and by diminishing ATP levels [25,28]. In human chondrocytes, SNP decreased the Δψm as measured by the ratio of red/green fluorescence of DePsipher and inhibited the activity of complex IV of the MRC [10]. Regulation of the MRC may be a signalling pathway by which NO modulates articular cartilage matrix biosynthesis and pathologic mineralization. There are no previous studies of mitochondrial integrity in relation to the energy production in synoviocytes, although some mtDNA somatic mutations and the importance of normal Δψm have been studied [29,19]. Because the synovial membrane is an aerobic tissue, we speculated that mitochondrial integrity is necessary for synoviocyte survival. Studies have shown that proteins that are clearly regulated by mitochondria, such as the bcl-2 family, regulate the viability of synoviocytes [30]. Previous studies also found that NO induced synoviocyte death by regulating the expression of the protein p53 and the activation of caspase 3, although we found no publications concerning the importance of the MRC in synoviocyte ATP production [31,32,19]. For these reasons, we set out to study the effects of NO in relation to mitochondrial function in human OA synoviocytes. In our study, the NO donor SNP was able to reduce the survival of synoviocytes, as expected. We observed that SNP increased the hypodiploid cell number (data not shown), similar to an apoptotic or similar programmed cell death, as other authors have elucidated [19,31]. We thought that this decrease in cell viability might be due in part to the effect of NO on mitochondrial function. From our results, we have concluded that SNP depolarized Δψm and, as a probable consequence of irregular electron distribution along this membrane, we found a reduction in ATP production. However, the activities of the MRC complexes I and III were increased, possibly due to an adaptive effort by damaged synovial cells to produce more energy. We also found a decrease in CS activity, indicating a reduction in the mitochondria mass following SNP treatment, probably causing the ATP depletion. Apart from mitochondrial dysfunction, another explanation for the lowered ATP levels could be that SNP reduces the expression of G3PDH protein, an important enzyme in glucose metabolism in OA synoviocytes [33]. It appears that SNP binds to glucose receptors irreversibly [33], impacting the glycolytic pathway and generation of energy. The low levels of ATP explain why the synoviocytes die. We also were able to show that the mcl-1 and bcl-2 anti-apoptotic proteins were downregulated in the presence of SNP after 48 hours of treatment, helping to explain the programmed cell death NO induces [34]. Another study has shown that mcl-1 has an important role in neuron survival before and after damage to DNA [35]. The full length of caspase 3 was also decreased, probably because it was transformed to the smaller activated state, as other investigators have shown [19]. Direct investigation of the functions of NO production by chondrocytes has been hampered by the lack of uniformity existing among different types of NO donor compounds employed in the experiments [36]. The majority of studies have used SNP for the NO donor compound, and results show that it reduces cell survival and induces cell death with the morphologic changes characteristic of apoptosis [10,19]. A second possible explanation for the finding that NO reduced cell survival in all these cell types may lie in the interaction of additional reactive oxygen species (ROS), possibly concomitantly produced as a consequence of NO itself [19]. Also, an increase in intracellular levels of ROS from increased activity of MRC complexes I and III provides a condition where the intracellular redox status of the cell could be altered [37,38]. In this case, the action of NO on Δψm would increase the production of ROS by mitochondria and also the cytotoxicity associated with NO and some reactive nitrogen species (RNS), such as peroxynitrite [19]. A recent study on peroxynitrite mediated-chondrocyte apoptosis found that the predominant mode of cell death involved calcium-dependent cysteine proteases, known as calpains, and that peroxynitrite induced mitochondrial dysfunction in cells leading to caspase-independent apoptosis [39]. This hypothetical elevation of ROS and RNS production can form nitrotyrosine complexes with other proteins, increasing and amplifiying the effects that NO alone has as shown in joint tissues from OA and RA patients [40-42].

## Conclusions

In this study we demonstrated and elucidated the important role that NO has in mitochondrial integrity, in energy production and in regulation of the survival of synoviocytes. This information helps us understand the role of mitochondria in the development of OA pathology.

## Abbreviations

ATP: adenosine triphosphate; CS: citrate synthase; IL: interleukin; iNOS: inducible nitric oxide synthase; LTB4: leucotrien B4; MRC: mitochondrial respiratory chain; mg: milligram; min: minute; mm: millimetre; mM: millimolar; NO: nitric oxide; RA: rheumatoid arthtritis; OA: osteoarthtritis; Prot: proteins; ROS: reactive oxygen species; PGE2: prostaglandin 2; SNP: sodium nitroprusside; TNF-α: tumor necrosis factor alpha; μg: micro gram; μl: micro liter; μM: micro molar.

## Competing interests

### Financial competing interests

In the past five years, we have not received any reimbursements, fees, funding, or salary from any organization that may in any way gain or lose financially from the publication of this manuscript.

There is no organization financing this manuscript (including the article processing charge).

We do not hold any stocks or shares in any organization that may in any way gain or lose financially from the publication of this manuscript.

We do not currently hold any patents nor are we applying for any patents relating to the content of the manuscript.

We have not received reimbursements, fees, funding, or salary from any organization that holds or has applied for patents relating to the content of the manuscript.

### Non-financial competing interests

There are no non-financial competing interests (political, personal, religious, ideological, academic, intellectual, commercial or any other) to declare in relation to this manuscript.

## Authors' contributions

BCP carried out the synoviocyte cultures, molecular genetic studies, western blot experiments, performed the statistical analysis and drafted the manuscript. MAM and JA measured the MRC complex activities in digitonin-permeabilized synoviocytes, determined the mitochondrial membrane potential, and measured synoviocyte ATP levels. MJLA participated in the design and coordination of the study. FJB conceived the idea of the study, and participated in its design and coordination. All authors read and approved the final manuscript.

## Pre-publication history

The pre-publication history for this paper can be accessed here:

http://www.biomedcentral.com/1471-2474/12/42/prepub
